# Protected areas are not effective for the conservation of freshwater insects in Brazil

**DOI:** 10.1038/s41598-021-00700-0

**Published:** 2021-10-28

**Authors:** Karina Dias-Silva, Thiago Bernardi Vieira, Felipe Ferraz Figueiredo Moreira, Leandro Juen, Neusa Hamada

**Affiliations:** 1 Programa de Pós-Graduação em Ecologia, Universidade Federal do Pará, Av. Perimetral, Guamá, Pará CEP 66075-110 Brazil; 2 Instituto Oswaldo Cruz, Laboratório de Biodiversidade Entomológica, Av. Brasil, 4365, Pavilhão Mourisco, Sala 214, Manguinhos, Rio de Janeiro, RJ CEP: 21040-360 Brazil; 3 Laboratório de Ecologia E Conservação, Instituto de Ciências Biológicas, Universidade Federal Do Pará, Av. Perimetral, Guamá CEP 66.075-110, Belém, Pará Brazil; 4 Instituto Nacional de Pesquisas da Amazônia-INPA, Av. André Araújo, 2936, Petrópolis, Manaus, Amazonas CEP 69067-375 Brazil

**Keywords:** Data processing, Ecology

## Abstract

Biodiversity conservation has faced many challenges, especially the conversion of natural areas that compete with use for agriculture, energy production and mineral extraction. This problem is further aggravated by lack of knowledge of the biodiversity that exists and the geographical distribution of different groups. The objectives of our study were to examine the distributional pattern of Gerromorpha diversity in Brazil, create a map of conservation priority areas, estimate the degree of protection that the current network of protected areas guarantees to this insect group, and identify the size thresholds in geographical distributions that would allow species to be protected. We used species occurrences from the Water Bugs Distributional Database, and we used 19 bioclimatic variables to build models of the potential distributions of species using the MaxEnt program. Using the potential model results, we calculated diversity metrics and overlapped them with the current state and federal “conservation units” (protected areas for biodiversity) in Brazil. Total beta diversity and turnover portions were separated into two faunistic groups, one in northern and the other in southern Brazil. The Amazon has higher beta diversity than what was predicted by the null models. We detected a positive relationship between species distribution area and occurrence in conservation units. Conservation units with less than 250 km^2^ do not protect Gerromorpha species. Our results reinforce the necessity of formulating new conservation strategies for this group, contemplating species with both restricted and ample distributions, because rare and specialist species are the most harmed by habitat reduction, given that they are more sensitive to environmental disturbance.

## Introduction

Biodiversity conservation has faced many challenges in the past few years, especially because of the speed with which natural areas have been converted to areas for agriculture, energy production, mineral extraction and real-estate development^1^. This challenge is even bigger when areas of economic interest overlap with areas that are important for biological conservation^2,3^, creating socio-economic conflicts. In addition, the definition of areas for conservation is usually intended for flag species, synergistic species or species with a social appeal^4,5^, leaving aquatic and terrestrial insects out of this planning^6^. One way of diminishing this problem is by creating and implementing conservation units to conciliate nature conservation with sustainable resource use, making human presence in protected areas compatible with biodiversity conservation. In Brazil there are 12 types of “conservation units” (CUs) (*Unidades de Conservação* in Portuguese), or protected areas for biodiversity^7^. The different types of CUs differ in the level of access permitted, some are like ecological sanctuaries and have effective protection, being restricted to scientific research; others are less restricted. Some unit types are less restricted and allow use for recreation (e.g., National Parks) and even for resource exploitation (Extractive Reserves). Generally, each protected area has rules regarding its use. These areas shelter a high diversity of species, ecosystem services and traditions. CUs provide biodiversity maintenance, and (in some cases) biodiversity recovery^7^. However, not all protection measures are effective because some CUs are inefficient as a result of the areas being selected arbitrarily based either on the empirical knowledge of researchers or by being placed in areas with difficult access, which limits competing interests for agriculture or for real-estate development. Selection may be done considering only the specific characteristics of a restricted biological group, usually only accounting for terrestrial biodiversity^8^. Brazil’s current CU network is insufficient and does not adequately protect biodiversity^6,9^.

The selection of priority areas for conservation must consider an area’s representativeness, irreplaceability, flexibility, complementarity and persistence^10^. Area selection should be based on biogeographical and ecologically georeferenced information, so as to permit analyses and interpretation at different scales^11^. However, it is often impossible to fully protect insect biodiversity due to the lack of biogeographical information on species^3,12^. In the Neotropical region the lack of knowledge is acute regarding real species distributions (the “Wallacean shortfall”)^12^. For instance, in Brazil, biogeographical information on species is scarce, even for biodiversity hotspots. Lack of information, together with the lack of financial resources and the overlapping interests between agricultural development and conservation are the main challenges in defining priority areas for conservation^6,13^. In addition, we do not know whether the current reserve network is sufficient to protect all the taxonomic groups. A study carried out on bats in the Cerrado showed that species with restricted distributions tend not to be covered by conservation units^14^. This result leads us to question the effectiveness of conservation units, especially for key species in ecosystems (such as aquatic insects, which are not ‘charismatic’).

Inefficiency of reserve networks may be related to non-protection of rare species^6^ or to choosing areas that are marginally adequate for species distributions^15^. One way of reducing inefficiency is by using other criteria for area selection, e.g., species richness, beta diversity, fauna complementarity and the number of endemic species^3,16^. These parameters, along with use of species-distribution models (SDMs) could reduce the problem of lack of knowledge about distributions that leads to protection of marginally adequate areas. This knowledge would allow selection of areas having high probability of occurrence of a species and that provide favorable environmental conditions. Although this approach has been shown to be robust for designing prioritization measures e.g.:^17–21^ the criteria used are generally based on a few groups of vertebrates and plants, which reduces the effectiveness of protected areas for conserving the biodiversity of all groups. Consequently, several biodiversity components, such as aquatic invertebrates, might not be included in the protected areas^6,9^.

In aquatic ecosystems, macroinvertebrates stand out from other groups because of their sensitivity to environmental impact^22,23^ and their role in nutrient cycling and energy transfer in food chains. Insects in the suborder Heteroptera are predators, are at the top of food chains, can respond to changes happening in lower trophic levels, and are considered to be good models for evaluating environmental impact^24^. Heteroptera occupy an ample variety of habitats, including waterbodies that are either lotic or lentic and either perennial or temporary; these insects have an important role in biological control in waterbodies^25–28^. Heteroptera is composed of three infraorders: Nepomorpha (truly aquatic), Gerromorpha and Lepdomorpha (both semi-aquatic). The infraorder Gerromorpha is divided into 20 families, 325 genera, and approximately 4700 species inhabiting freshwater ecosystems: 28% of these species are in the Neotropics^29^. Most Gerromorpha species live over the water column, either on floating plants or between plant roots on the edges of freshwater bodies^30^. Thus, they are affected by forest removal and increased waterflow in streams^27,31^. Approximately 2100 species have been described for Gerromorpha in the Neotropics, which currently has eight families and about 160 genera^29^, of which 208 species and subspecies occur in Brazil^32^. This group was chosen because it occupies various types of aquatic ecosystems, has an ample geographical distribution, and perhaps acts as an umbrella or surrogate group encompassing additional aquatic species that inhabit the same sites^33,34^.

Due to the speed with which natural areas have been modified by anthropogenic activities and the lack of knowledge about the state of conservation of aquatic insects, our objectives are to: (i) describe distributional patterns of Gerromorpha diversity (Richness and Beta Diversity) among Brazilian biomes; (ii) create a map of conservation priority areas for this group; (iii) assess the importance of the current network of conservation units and (iv) identify the threshold for the extent of geographical distribution needed to protect species under the current Brazilian system of conservation units.

## Results

Of 208 Gerromorpha species recorded in Brazil, we found 3541 occurrences and modeled 111 species (only species with three or more occurrences are suitable for this procedure) (Table S1). The Monte Carlo simulation tests to evaluate the dependency of AUC on the number of occurrences were not significant for all classes (Table 1), which means that there is no relationship between the number of occurrences and the AUC values obtained from the model^35^.

**Table 1:** Values of Monte Carlo tests performed to identify the relationship between the numbers of occurrences and the observed AUC values.

Number of occurrences	Number of species	AUC mean	*p*
4 to 15	76	0.915	
16 to 30	18	0.954	0.63
31 to 45	10	0.951	0.82
46 to 60	2	0.918	0.52
61 to 75	4	0.902	0.96

*AUC* area under the curve.

**Figure 1:**
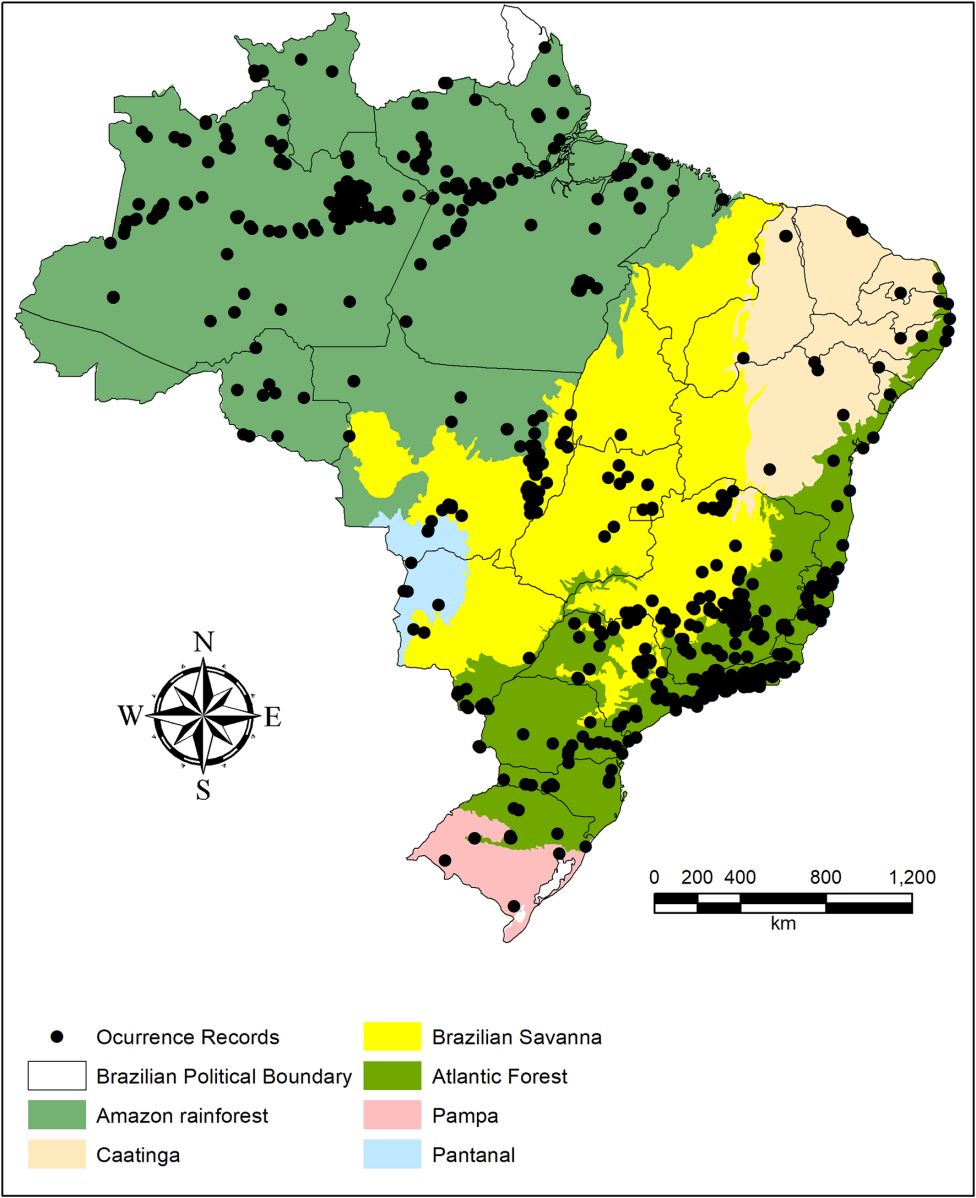
Gerromorpha species occurrences used in the species distribution models (SDM). The figure was built on the R environment^63^.

**Figure 2:**
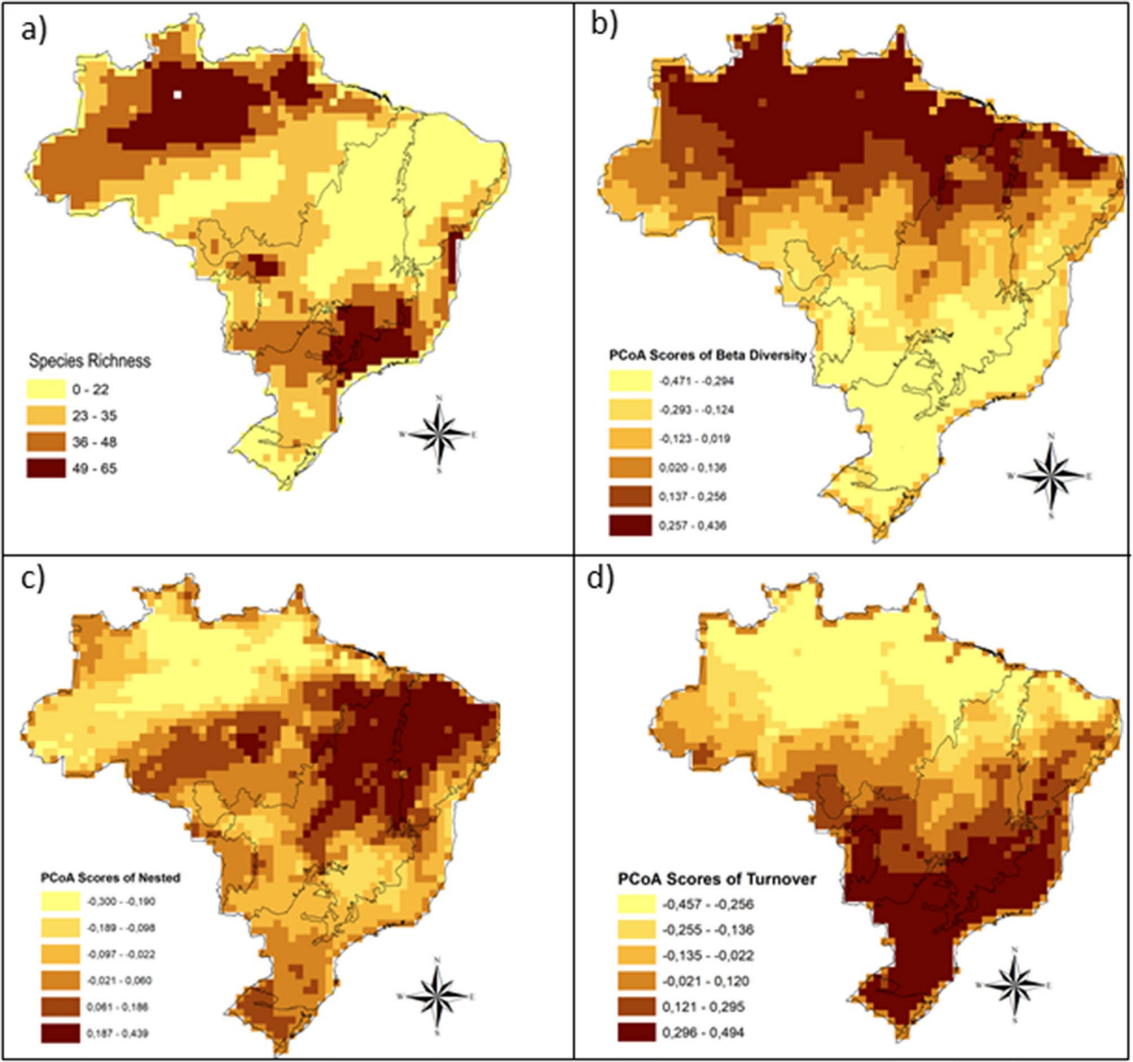
Predicted spatial distributions, based on species predicted presences resulting from the binarization of MaxEnt output of (**a**) richness, (**b**) total beta diversity, (**c**) turnover and (**d**) nestedness of Gerromorpha in Brazil. The figure was built on the R environment^63^.

Significant portions of the species-occurrence sites were in the Southeastern Region of Brazil, in the Atlantic Forest biome, and in the Northern Region (Amazon) (Fig. 1). The areas with the highest richness were the northern portion of the Amazon, eastern and western Cerrado (central Brazilian savanna), and a small coastal portion in the northern Atlantic Forest (Fig. 2a). The Atlantic Forest and the Amazon had higher richness than predicted based on random sampling of grid cells (Table 2). When evaluating total beta diversity, the highest values were found for the Northern Region of Brazil (Fig. 2b), and the Amazon biome had higher beta diversity than predicted (Table 2). However, when evaluating the components of beta diversity, the turnover portion (Fig. 2d) was greatest in the Southern Region of Brazil (Fig. 2d). On the other hand, in the nested portion we found the areas with the lowest species richness values (the northern Cerrado and Caatinga, as well as the central Amazon); these are the areas with the highest nestedness values for the Gerromorpha (Fig. 2c).

**Table 2:** Random and observed mean (M) and standard deviation (SD) values of Gerromorpha richness and total beta diversity in Brazilian biomes. N—number of observed cells in each biome; p—significance value according to the Monte Carlo randomization test.

Biome	N	Richness			Beta diversity		
		Observed (M ± SD)	Random (M ± SD)	*p*	Observed (M ± SD)	Random (M ± SD)	*p*
Amazon	998	36 ± 12	32 ± 1	< 0.001	0.159 ± 0.177	< 0.001 ± 0.005	< 0.001
Caatinga	231	17 ± 8	32 ± 2	0.984	0.030 ± 0.181	< 0.001 ± 0.150	0.024
Cerrado	468	29 ± 14	32 ± 1	0.893	(−)0.185 ± 0.211	< 0.001 ± 0.010	0.074
Atlantic Forest	194	38 ± 11	32 ± 1	< 0.001	(−)0.317 ± 0.143	< 0.001 ± 0.017	0.872
Pampas	57	22 ± 5	32 ± 2	0.072	(−)0.220 ± 0.170	< 0.001 ± 0.033	0.032
Pantanal	21	30 ± 3	32 ± 4	0.771	(−)0.273 ± 0.137	< 0.001 ± 0.056	0.023

**Figure 3:**
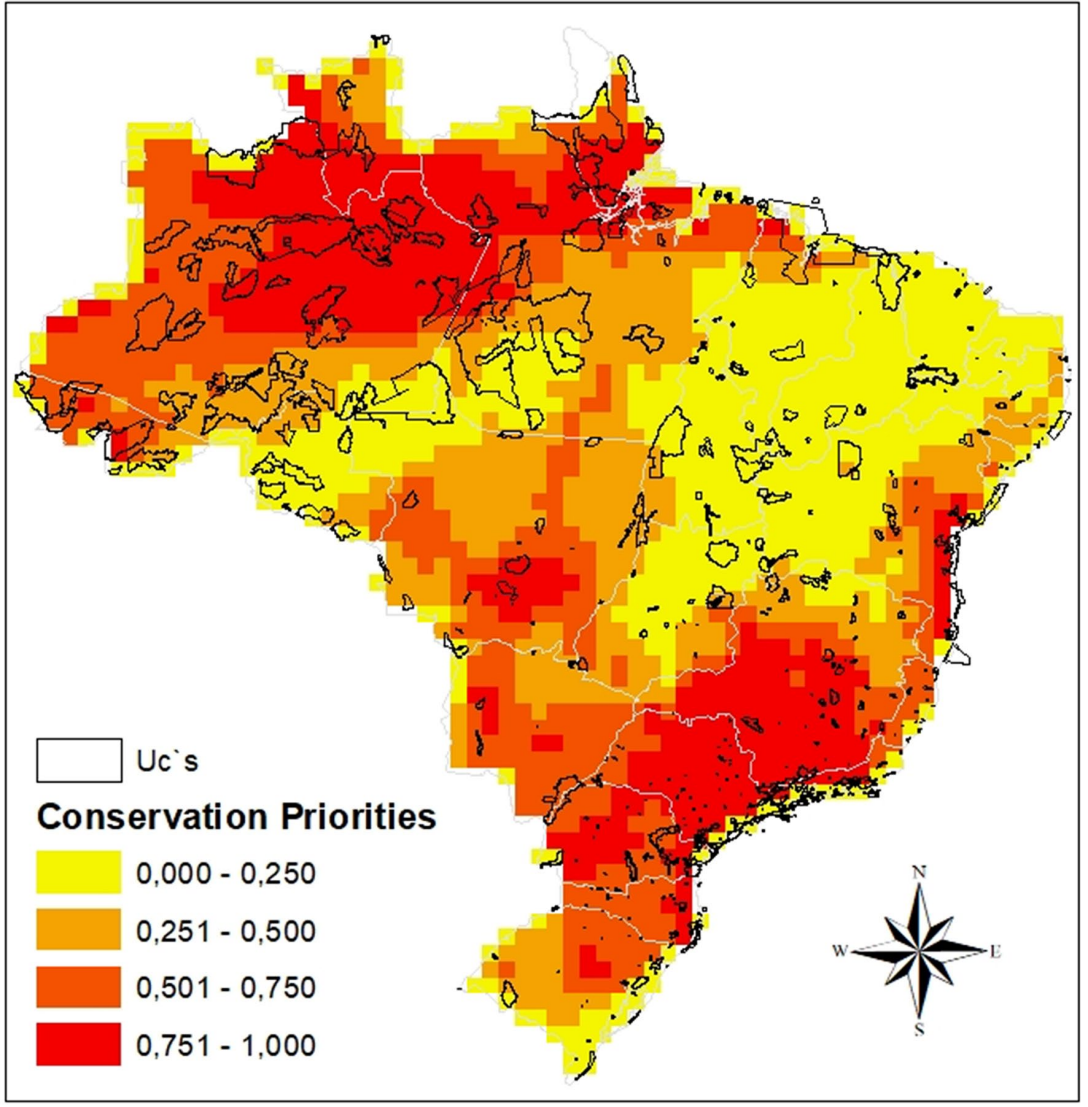
Priority areas for Gerromorpha conservation in Brazil, according to the Zonation algorithm. Values indicate the importance of each cell; the higher the value, the higher the importance. The figure was built on the R environment^63^.

**Figure 4:**
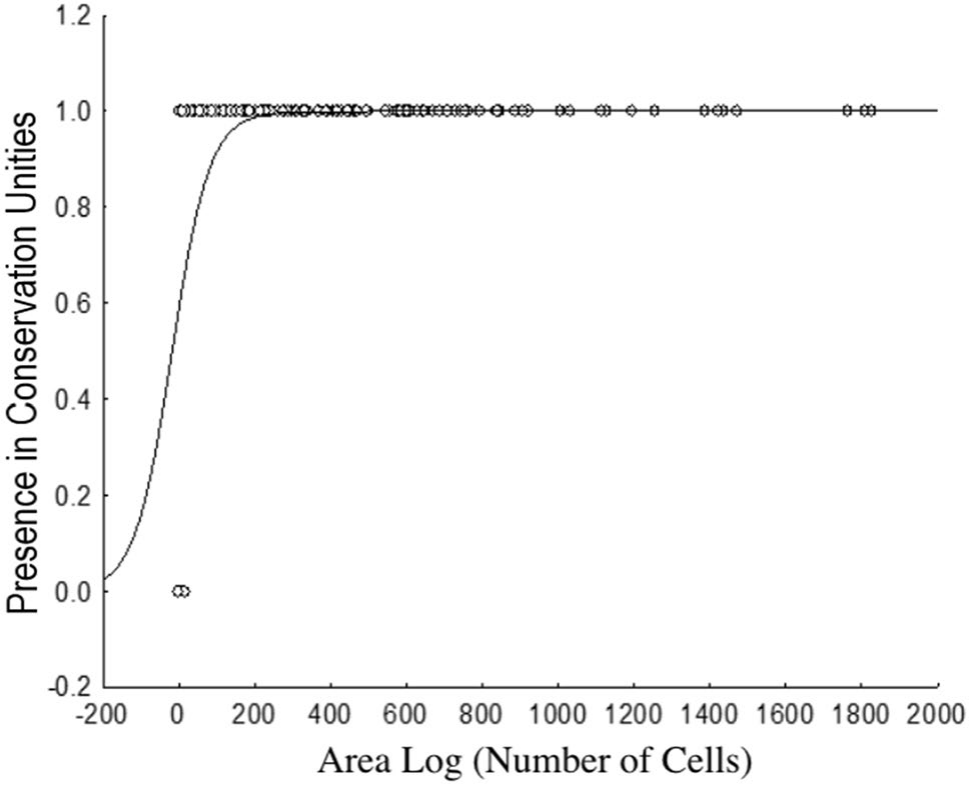
Logistic relationship between Gerromorpha occurrence and presence of these species in CUs. Four species are located outside the protected area of the biome, where zero (0) means location outside a CU and one (1) means presence in a CU.

The map of cell importance for conservation (Fig. 3) shows that cells in the northern Amazon, the eastern and western Cerrado and in the coastal portion of the Atlantic Forest are important for Gerromorpha conservation, and many locations that are considered priority areas are outside of the conservation units. In general, the biomes did not differ in terms of importance for the conservation of Gerromorpha (Table 3), and we observed for both the general model (evaluating conservation units in Brazil as a whole) and the regional models (evaluating the conservation units in each biome) that conservation units do not protect either more or less areas that are important for Gerromorpha than what was randomly expected (Table 3). Lastly, we observed a positive relationship between species distribution areas and species occurrences in conservation units (x^2^_(1)_ = 36.144 *p* < 0.001). The minimum occurrence for a species to be present within a conservation unit is approximately five cells, i.e., 250 km^2^ (Fig. 4).

**Table 3:** Random and observed mean (M) and standard deviation (SD) of importance values for the conservation of Gerromorpha in Brazil (Total) and for each biome. N—number of observed cells in each biome; NUC—number of protected cells in each biome; p—significance value according to the Monte Carlo randomization test.

Biome	N	Conservation unit				Biome		
		NUC	Observed (M ± SD)	Random (M ± SD)	*p*	Observed (M ± SD)	Random (M ± SD)	*p*
Total	1969	262	0.462 ± 0.097	0.503 ± 0.066	0.811			
Amazon	998	188	0.509 ± 0.082	0.502 ± 0.019	0.344	0.502 ± 0.081	0.502 ± 0.000	0.965
Caatinga	231	16	0.208 ± 0.053	0.174 ± 0.043	0.210	0.174 ± 0.032	0.174 ± 0.002	0.986
Cerrado	468	34	0.361 ± 0.085	0.455 ± 0.051	0.966	0.455 ± 0.096	0.455 ± 0.003	0.586
Atlantic Forest	194	19	0.492 ± 0.202	0.624 ± 0.062	0.977	0.623 ± 0.082	0.624 ± 0.004	0.987
Pampas	57	4	0.118 ± 0.056	0.294 ± 0.119	0.916	0.294 ± 0.060	0.294 ± 0.014	0.978
Pantanal	21	1	0.549	0.498 ± 0.259	0.985	0.501 ± 0.070	0.498 ± 0.067	0.264

## Discussion

Cells protected by conservation units (CUs) have a lower degree of importance than expected, based on their values of complementarity and irreplaceability resulting from application of the zonation algorithm. This low importance, determined according to Gerromorpha biodiversity, may be related to the fact that the CUs have been created arbitrarily, probably based on economic interest or to protect specific taxonomic groups^3^ that are not aquatic^9^. These results indicate that the current network of CUs does little to protect Gerromorpha species, especially those with restricted distributions, and especially species with distributions smaller than 250 km^2^. CUs may be protecting sites with marginal environmental suitability for the occurrence of these species e.g.:^6,14,36^, thereby making these species more susceptible to environmental changes, such as those generated by anthropogenic activities and climate change. These results support the conclusion that conservation strategies should be based on conservation policies and not only on the productive value of the areas (which are generally areas with low agricultural interest), or based on political considerations^37^. When decisions on protected areas are based solely on economic and political interests they reduce the possibility of including species with restricted distributions or that occur in areas with high economic value for agricultural production^10,14,38–41^.

Creating new conservation units is a slow process because it depends on financial resources to buy land, provide area maintenance and pay teams working in the unit, besides the various impediments imposed by the conflicts-of-interest mentioned earlier. Thus, an economically viable possibility would be to maintain existing forest fragments and encourage farmers to maintain legal reserves and riparian vegetation. Forest remnants and legal-reserve areas would act as stepping stones for biodiversity, supporting the maintenance of connectivity among CUs. In addition, healthy riparian vegetation keeps catchments fully preserved, further enhancing connectivity among forest remnants, legal reserves and CUs. Finally, we emphasize that, although the maintenance of riparian forests is considered to be sufficient for maintaining the Gerromorpha community in running waters, other strategic areas (such as those presented in this paper) must be maintained in addition to the riparian strips. Otherwise one risks only species with wide occurrence ranges will be preserved, leaving rare and short-range endemics outside the network of CUs.

In terms of species richness, we found that the Atlantic Forest and the Amazon biomes shelter the highest number of Gerromorpha species per unit area, and the richness in these biomes is higher than would be expected if random, suggesting that their habitats are more heterogeneous than in the other Brazilian biomes. This idea is supported by the results on total beta diversity, suggesting that the Gerromorpha composition in these biomes results from steep environmental gradients. On the other hand, we observed the Pampas biome to have fewer species than expected, suggesting that these areas have low heterogeneity for this group.

Regarding priority areas for Gerromorpha sampling, we observed that all biomes have low sampling rates. Some examples are the “arc of deforestation” in the southern portion of the Amazon biome, large parts of the Pantanal and Caatinga biomes, and part of the Atlantic Forest biome. In general, we observed that most sampling is concentrated near institutions. If we consider the vast river network in Brazil we can conclude that the number of records we have is small, providing an example of the “Wallacean shortfall”^42^, where information about species distributions is still insufficient. Additional sampling is needed so that information on species distributions can be expanded and more occurrence sites can be identified, enabling the discovery of new species and the generation of information on species distributions to assist in choosing conservation areas^39^. We also emphasize the importance of evaluating the extreme northern portion of the Amazon and the southeastern Cerrado, which are very relevant for conservation and have high species richness but are not well studied. Furthermore, the southeastern portion of the Cerrado stands out for its large turnover component, suggesting a strong environmental filter, possibly due to the existence of small fragments of vegetation, that can be used as stepping stones by some species (few in this case) but that cannot be used as habitat. The northern portion of the Cerrado and the Caatinga stand out for their nesting components, suggesting that species dispersion is easily observed within and between these biomes, as observed for bats in the Cerrado^14^. The nesting component was justified by the existence of large preserved areas in the northern portion of the Cerrado biome, which has favored the mobility of bats^14^. The turnover component was justified by the local extinction of some species because the southern and southwestern portions of the Cerrado are highly fragmented, which hampers dispersal and may cause local extinctions of species. The high diversity of Gerromorpha in these regions is also congruent with other biological groups, e.g., birds, reptiles, amphibians and non-flying mammals^43,44^. Hence, by preserving the diversity of Gerromorpha, we are also preserving the diversity of other groups, and ensuring self-sustaining ecosystems, thus being able to recover from stochastic events or even long-term climate change. We therefore stress that CUs defined with little or no planning have low importance for Gerromorpha conservation and do not sufficiently preserve species with restricted distributions (less than 250 km^2^ for Gerromorpha). New strategies for the conservation of this group are needed, especially addressed to conserve both species with restricted ranges and those with broad distributions^6^ because rare and specialist species are the most affected by habitat reduction and are the most sensitive to environmental disturbance.

## Materials and methods

### Species occurrence data

Species occurrences (Fig. 1) were obtained from the Water Bugs Distributional Database (available at https://sites.google.com/site/distributionaldatabase/). This database represents an extensive research effort to obtain information from the literature. The database contains occurrence information for all Gerromorpha species known in Brazil together with the sources from which the information was obtained. More information about the database is available Dias-Silva et al.^45^.

Initially, coordinates of the occurrence data were transformed from the original geographic reference system into decimal degrees (in datum SAD 69), using the SpeciesLink website (available http://splink.cria.org.br/conversor?criaLANG=pt). This transformation is necessary to perform the SDMs because the algorithms only recognize decimal degree as geographic coordinates. In order to avoid any bias caused by geographically imprecise data, we eliminated occurrences where the distance between point coordinates and the seat of the municipality was less than 2 km^46,47^.

### Environmental variables

We used 19 bioclimatic variables to build the models; the values were obtained from monthly data on temperature and precipitation available on the WorldClim 1.4. platform^48^. The climatic variables have a resolution of approximately 9 km (≈ 0.083 decimal degrees) and are highly colinear. In order to solve or reduce the collinearity problem, we performed a principal component analysis (PCA)^49^, from which we extracted seven axes that explained, in total, 95% of the variation in the original dataset. These axes were used as predictive environmental variables in the potential species distribution model (SDM). We tried therefore to reduce the multicollinearity problem of the original variables, making it possible to model species distributions with a small dataset on occurrence^50^.

### Distribution models and model evaluation

Potential species distributions were estimated using the MaxEnt (Maximum Entropy Modeling) program version 3.3.3^51^. To analyze the performance of the potential species distribution model, we used two techniques: AUC (area under the curve) and TSS (true skilled statistics). The AUC measure, which is the area under the ROC (receiver operating characteristic) curve, has scores varying between 0 and 1, where values ≈ 0.5 (or smaller) correspond to models predicting no better than randomly fitted ones, and values ≥ 0.7 are considered acceptable^52,53^. True skilled statistics (TSS) scores vary from -1 to 1, where scores close to zero and negative scores are no better than random scores, and scores close to 1 have a perfect fit between the observed distribution and the predicted distribution^54^. Values ≥ 0.5 indicate acceptable models in the field of ecology and for the purpose of protected-area design e.g.:^14,19,20,55–57^. To convert predicted species distributions into presence/absence maps, we used threshold values^58,59^ that were derived from the ROC curve. Use of the maximum threshold value (the Max TSS threshold) to maximize the specificity and sensitivity of models and tends to produce more-restricted distributions^19^, e.g.:^57,60,61^. Additionally, we performed a Monte Carlo simulation test to evaluate the dependency of AUC on the number of occurrences^6^ (Table 1). Thus, we divided the data into five classes (with an interval of 15 locations between the classes). For each class we calculated the mean of the observed AUC (sum of the AUCs of all models in the class, divided by the number of species present in the class) (Table 1). The first class (4–15 occurrences) was considered as the control, using the observed mean AUC as a critical value for all other classes. For AUC value simulations we maintained the species number inside the classes equal and calculated the mean AUC of a random set of species, called the “random AUC mean.” For this we choose a random set of species (the number of species selected was the same as the number of species present in the class) and calculated the random AUC mean. This procedure (random AUC mean) was repeated 10,000 times, and random AUC mean values greater than or equal to the observed AUC mean of the control class (between 4 and 15 occurrences) were counted. This number was finally divided by 10,000 to assess the p-value of the class. The null hypotheses in our analysis was that there is no relationship between an increasing number of occurrences and an increasing AUC value. This procedure was repeated for all classes, and the results were not significant.

### Data analysis

To identify the spatial distribution of Gerromorpha diversity in Brazil, we used a grid with a resolution of 0.083° (the same resolution as the SDM) and extracted information on species presence and absence from potential distribution maps. Therefore, each row represents a grid cell and each column represents a species, and the spreadsheet cells represent species presence or absence. This table summarizes the predicted presence or absence for each target species in the Brazilian territory. Using this table, we calculated the total species richness of each cell based on the sum of species presences along the corresponding row and on the beta diversity. For beta diversity we used the procedure described by Baselga et al.^62^, in which diversity is partitioned into portions for nestedness and turnover, and the sum of these portions represents total beta diversity. For the spatialization of beta diversity (the total of the nested and turnover portions), we performed a principal coordinates analysis (PCoA)^49^ on each diversity matrix, and the score of the first PCoA axis was used as a synthesis of the diversity for the cell and is shown on a map (one map for each component). Thus, we obtained values for richness, total beta diversity, nested beta diversity and turnover beta diversity for each grid cell. This allowed us to spatialize diversity values by categorizing the cells into color gradients. This procedure was performed for each of the diversity measures.

We used a Monte Carlo randomization procedure with 10,000 randomizations to evaluate diversity distribution among Brazilian biomes. For this procedure we developed a routine in the R environment that classifies grid cells according to the biome to which cells belong and calculates the mean and standard deviation of the diversity metrics for each biome. We chose cells randomly for the significance test (always controlling the number of observed cells in the biome) and calculated the mean random diversity. For this we assessed the number of cells present in each biome (See Table 2) and calculated the mean and standard deviation of the diversity metrics of these cells. We then selected random cells (the same number present in the focal biome) and calculated the random mean and standard deviation of the diversity metrics of these cells. This procedure was repeated 10,000 times end for each biome. To calculate the significance value, we identified the number of random values higher than the observed values and divided them by 10,001. This procedure was performed for richness and total beta diversity.

To determine priority areas for conservation of aquatic species in Brazil, we used the Zonation algorithm^63^, along with data on the potential distribution of target group. Zonation is a quantitative method that prioritizes conservation areas, aiming to long-term biodiversity persistence^64^. Evaluation is done by randomly removing cells. This analysis can be performed using three algorithms: Core-area Zonation, Additive benefit function and Target-based planning. For this study, we used the additive-benefit function because it is best suited for a high number of species. This analysis is run with the complete landscape, where sites are classified based on biological values (complementarity and irreplaceability), and subsequently the less-valuable cells are removed one (or more) at a time, producing a sequence of landscape structures with more resources for biodiversity^65^.

To evaluate the difference in importance between protected and unprotected cells, we used a second routine in the R environment based on the Monte Carlo randomization test with 10,000 randomizations. Cells were classified into those that were “protected” (if they were totally or at last 50% within a conservation unit) and those that were “unprotected,” and the mean importance (accessed from the Zonation analysis) of protected cells was estimated. The same number of unprotected cells was selected randomly, and we estimated the mean importance. The estimate of the random importance value was calculated 10,000 times, thus creating the expected distribution of the importance values. This procedure was performed for the reserve network of Brazil as a whole and for the reserve network inside each biome. The significance value was obtained by dividing the number of values that were greater than or equal to the observed values by 10.001^66^. The reserve network considered for the analyses was the official map of conservation units in the “full protection” category, which is available in the Brazilian Ministry of Environment (MMA) website (http://mapas.mma.gov.br/i3geo/datadownload.htm). The official biome borders (also available on the MMA website) were considered to represent the historical distribution of the biological biomes.

To evaluate if there is a minimum range size for a species to be protected by a conservation unit, we performed a logistic regression between the potential extent of occurrence as predicted by the binarized SDMs for species distribution areas and their presence/absence inside the units. Species distribution area was estimated using the sum of the numbers of cells in which the species occurs, and the cells were classified as being inside (1) or outside (0) of conservation units. A non-linear logistic regression was performed^35,67^, where presence/absence was considered to be the dependent variable.

## Supplementary Information

Supplementary Information.

## Data Availability

All occurrences data are available in Water Bugs Distributional Database (https://sites.google.com/site/distributionaldatabase/).
